# Pandemic preparedness and health system resilience in 14 European countries

**DOI:** 10.2471/BLT.23.290509

**Published:** 2024-06-10

**Authors:** Kaitlyn Hall Radford, Marina Karanikolos, Jonathan Cylus

**Affiliations:** a Faculty of Public Health and Policy, London School of Hygiene and Tropical Medicine, Keppel Street, London WC1E 7HT, England.; b European Observatory on Health Systems and Policies, London School of Hygiene and Tropical Medicine, London, England.; c European Observatory on Health Systems and Policies, London School of Economics and Political Science, London, England.

## Abstract

**Objective:**

To assess national pandemic preparedness and response plans from a health system perspective to determine the extent to which implementation strategies that support health system performance have been included.

**Methods:**

We systematically mapped pandemic preparedness and response implementation strategies that improve resilience to pandemics onto the Health System Performance Assessment Framework for Universal Health Coverage. Using this framework, we conducted a document analysis of 14 publicly available national influenza pandemic preparedness plans, submitted to the European Centre for Disease Prevention and Control, to assess how well health system functions are accounted for in each plan.

**Findings:**

Implementation strategies found in national influenza pandemic preparedness plans do not systematically consider all health system functions. Instead, they mostly focus on specific aspects of governance. In contrast, little to no mention is made of implementation strategies that aim to strengthen health financing. There was also a lack of implementation strategies to strengthen the health workforce, ensure availability of medical equipment and infrastructure, govern the generation of resources and ensure delivery of public health services.

**Conclusion:**

While national influenza pandemic preparedness plans often include provisions to support health system governance, implementation strategies that support other health system functions, namely, resource generation, service delivery, and in particular, financing, are given less attention. These oversights in key planning documents may undermine health system resilience when public health emergencies occur.

## Résumé

**Objectif:**

Évaluer les plans nationaux de préparation et de réaction aux pandémies du point de vue du système de santé afin de déterminer le niveau d’inclusion des stratégies de mise en œuvre qui soutiennent la performance du système de santé.

**Méthodes:**

Nous avons systématiquement identifié les stratégies de mise en œuvre de la préparation et de la réaction aux pandémies qui améliorent la résilience dans le cadre d’évaluation de la performance des systèmes de santé pour la couverture sanitaire universelle. Ce cadre nous a servi de base pour procéder à une analyse documentaire de 14 plans nationaux de préparation à une pandémie de grippe accessibles au public. Ces plans ont été soumis au Centre européen de prévention et de contrôle des maladies, afin d’évaluer la prise en compte des fonctions des systèmes de santé dans chaque plan.

**Résultats:**

Les stratégies de mise en œuvre figurant dans les plans nationaux de préparation aux pandémies de grippe ne prennent pas systématiquement en compte toutes les fonctions des systèmes de santé. Elles se concentrent plutôt sur des aspects spécifiques de la gouvernance. En revanche, les stratégies de mise en œuvre visant à renforcer le financement de la santé ne sont que peu, voire pas, mentionnées. Les stratégies de mise en œuvre visant à renforcer les effectifs du personnel de santé, à garantir la disponibilité des équipements médicaux et des infrastructures, à régir la génération de ressources et à assurer la prestation de services de santé publique font également défaut.

**Conclusion:**

Si les plans nationaux de préparation aux pandémies de grippe comprennent souvent des dispositions visant à soutenir la gouvernance des systèmes de santé, les stratégies de mise en œuvre qui soutiennent d’autres fonctions des systèmes de santé, notamment la génération de ressources, la prestation de services et, en particulier, le financement, bénéficient d’une attention moindre. Ces lacunes dans des documents de planification clés sont de nature à nuire à la résilience du système de santé en cas de situation d’urgence liée à la santé publique.

## Resumen

**Objetivo:**

Evaluar los planes nacionales de preparación y respuesta ante una pandemia desde la perspectiva del sistema sanitario para determinar en qué medida se han incluido estrategias de implementación que apoyen el rendimiento del sistema sanitario.

**Métodos:**

Se trazó un mapa sistemático de las estrategias de preparación y respuesta ante pandemias que mejoran su resiliencia en el Marco de Evaluación del Rendimiento del Sistema Sanitario para la Cobertura Sanitaria Universal. A partir de este marco, se realizó un análisis documental de 14 planes nacionales de preparación ante una pandemia de gripe y de acceso público, presentados al Centro Europeo para la Prevención y el Control de las Enfermedades, con el fin de evaluar en qué medida se tienen en cuenta las funciones del sistema sanitario en cada plan.

**Resultados:**

Las estrategias de implementación que se encuentran en los planes nacionales de preparación ante una pandemia de gripe no consideran sistemáticamente todas las funciones del sistema sanitario. En su lugar, se centran sobre todo en aspectos específicos de la gobernanza. Por otro lado, apenas se mencionan las estrategias de aplicación destinadas a reforzar la financiación sanitaria. También faltan estrategias de implementación para reforzar el personal sanitario, garantizar la disponibilidad de equipos médicos e infraestructuras, gobernar la generación de recursos y garantizar la prestación de servicios sanitarios públicos.

**Conclusión:**

Aunque los planes nacionales de preparación ante una pandemia de gripe suelen incluir disposiciones para apoyar la gobernanza del sistema sanitario, se presta menos atención a las estrategias de implementación que apoyan otras funciones del sistema sanitario, en concreto, la generación de recursos, la prestación de servicios y, en particular, la financiación. Estos descuidos en documentos de planificación clave pueden debilitar la resiliencia del sistema sanitario cuando se producen emergencias de salud pública.

## ملخص

**الغرض:**

تقييم خطط الاستعداد الوطني للجوائح والاستجابة لها، من منظور النظام الصحي، وذلك لتحديد درجة إدراك استراتيجيات التنفيذ التي تدعم أداء النظام الصحي.

**الطريقة:**

لقد اتبعنا أسلوبًا منهجيًا في تخطيط استراتيجيات الاستعداد للأوبئة وتنفيذ الاستجابة لها، تلك الاستراتيجيات التي تعمل على تحسين المرونة تجاه الأوبئة في إطار عمل تقييم أداء النظام الصحي من أجل التغطية الصحية الشاملة. وباستخدام إطار العمل هذا، قمنا بإجراء تحليل للوثائق في 14 خطة وطنية متاحة للعامة للاستعداد لجائحة الأنفلونزا، وهي خطط تم تقديمها للمركز الأوروبي للوقاية من الأمراض ومكافحتها، بهدف تقييم مدى جودة الاهتمام بوظائف النظام الصحي في كل خطة.

**النتائج:**

إن استراتيجيات التنفيذ الموجودة في الخطط الوطنية للاستعداد لجائحة الأنفلونزا، لا تضع في اعتبارها كل وظائف النظام الصحي بشكل منهجي. بل إنها تركز في الأغلب على جوانب محددة من الإدارة، بدلا من ذلك. وعلى وجه النقيض، لم يتم ذكر سوى القليل من استراتيجيات التنفيذ التي تهدف إلى دعم تمويل الصحة، أو لم يتم ذكرها من الأساس. كما كان هناك أيضًا نقص في استراتيجيات التنفيذ الساعية لدعم القوى العاملة في القطاع الصحي، وضمان توفر المعدات الطبية والبنية التحتية، وإدارة توليد الموارد، وضمان تقديم خدمات الصحة العامة.

**الاستنتاج:**

في حين أن الخطط الوطنية للاستعداد لجائحة الأنفلونزا غالباً ما تتضمن بنودًا لدعم إدارة النظام الصحي، إلا أن استراتيجيات التنفيذ التي تدعم وظائف النظام الصحي الأخرى، أي توليد الموارد وتقديم الخدمات، وعلى وجه التحديد التمويل، تحظى بقدر أقل من الاهتمام. قد تؤدي هذه الحالات من الإهمال في وثائق التخطيط الرئيسية إلى التقليل من أهمية مرونة النظام الصحي عند حدوث حالات طوارئ في الصحة العامة.

## 摘要

**目的:**

从卫生系统的角度评估各国大流行病的防范和应对计划，以确定支持卫生系统绩效的实施战略的纳入程度。

**方法:**

我们将可以提高大流行病抵御能力的大流行防范和应对实施战略系统地映射至旨在实现全民健康覆盖的卫生系统绩效评估框架中。我们根据该框架对提交给欧洲疾病预防和控制中心的 14 个可公开获取的全国大流行性流感防范计划进行了文档分析，以评估在每个计划里卫生系统职能的受重视程度。

**结果:**

我们发现，各国大流行性流感防范计划中列出的实施战略未能系统地考虑到所有卫生系统职能。相反，这些战略主要侧重于具体的治理措施。相比之下，很少甚至几乎没有计划提及旨在加强卫生筹资的实施战略。同时，还缺乏加强卫生工作人员队伍建设、确保医疗设备和基础设施可用、管理资源生成以及确保公共卫生服务到位的实施战略。

**结论:**

虽然各国大流行性流感防范计划通常都包含支持卫生系统治理的条款，但对支持其他卫生系统职能（即资源生成、确保服务到位以及特别是资金筹措）的实施战略却不太重视。当发生突发公共卫生事件时，关键规划文件中出现的这类疏忽可能会削弱卫生系统的复原力。

## Резюме

**Цель:**

Оценить национальные планы по обеспечению готовности к пандемии и реагированию на нее с точки зрения системы здравоохранения, чтобы определить масштабы включения в них стратегий реализации, способствующих повышению эффективности работы системы здравоохранения.

**Методы:**

Авторы систематически выполняли соотнесение стратегий обеспечения готовности к пандемиям и ответных мер, которые повышают устойчивость к пандемиям, с рамочной структурой системы оценки эффективности системы здравоохранения для всеобщего охвата услугами здравоохранения. На основе этой рамочной структуры был проведен анализ документов 14 общедоступных национальных планов готовности к пандемии гриппа, представленных в Европейский центр профилактики и контроля заболеваний, на предмет оценки полноты учета функций системы здравоохранения в каждом плане.

**Результаты:**

Стратегии реализации, содержащиеся в национальных планах готовности к пандемии гриппа, не учитывают систематически все функции системы здравоохранения. Напротив, в основном они сосредоточены на конкретных аспектах управления. В то же время практически не упоминаются стратегии реализации, направленные на укрепление финансирования здравоохранения. Также отсутствовали стратегии реализации, направленные на укрепление кадрового потенциала здравоохранения, обеспечение наличия медицинского оборудования и инфраструктуры, управление процессом генерирования ресурсов и обеспечение предоставления услуг общественного здравоохранения.

**Вывод:**

В то время как национальные планы готовности к пандемии гриппа часто включают положения, направленные на поддержку управления системой здравоохранения, стратегиям реализации, поддерживающим другие функции системы здравоохранения, а именно генерирование ресурсов, предоставление услуг и, в частности, финансирование, при этом уделяется меньше внимания. Эти недоработки в ключевых документах по планированию могут подорвать устойчивость системы здравоохранения при возникновении чрезвычайных ситуаций в области общественного здравоохранения.

## Introduction

The coronavirus disease 2019 (COVID-19) pandemic profoundly affected health systems as well as economies and societies more broadly.^1^^,^^2^ As countries grappled with increasing numbers of COVID-19 cases, many health systems struggled, not only to treat COVID-19 patients, but also to maintain access to non-COVID 19 services, leading to increases in waiting times and poor population health outcomes.^3^ The challenges faced by health systems during the pandemic raise important questions about the extent to which health systems are able to function during public health emergencies, such as a pandemic, and whether there are ways to better prepare them to ensure their sustained performance.

The concept of health system resilience has become increasingly important since the COVID-19 pandemic. Resilience is defined as “the ability to prepare for, manage (absorb, adapt and transform) and learn from a shock.”^4^ A wide range of well documented policies and pandemic preparedness and response implementation strategies (hereafter called implementation strategies) are available that can help health systems maintain their performance and continue meeting their main objective of improving health during a pandemic.^5^ The ability to put in place and act on these implementation strategies is a key determinant of a well performing, resilient health system during a crisis.^6^

Preparedness is a key component of resilience and implies that a well performing health system is forward-looking and able to plan for a possible shock.^6^ Pandemic preparedness plans are important documents at the national and international level that lay out the implementation strategies required to plan for and respond to large-scale infectious disease outbreaks.^7^ These plans are designed to limit the human, economic and societal consequences of emergencies; in practice they also have a strategic role in formulating policy actions. Therefore, pandemic preparedness plans need to consider health systems as a whole, and should not focus solely on containing an outbreak.

The World Health Organization (WHO) Strategic Framework for Emergency Preparedness, issued in 2017, includes resilient health systems as a key component of its multisectoral approach to support more timely and effective responses.^8^ However, at the same time, monitoring and evaluation tools, such as WHO’s States Party Self-Assessment Annual Reporting^9^ and Joint External Evaluation,^10^ do not account for the multidimensional effects that pandemics have on health systems, nor do they consider the extent to which health system-wide implementation strategies have been prepared to support resilient policy responses. Instead, these tools focus on a more limited set of actions, including countries’ obligations under the International Health Regulations (IHR) (2005)^11^ on notification and surveillance systems. As a result, investments in global health security may target selected areas and fail to factor in the importance of health system strengthening as a means of pandemic preparedness. Evaluation frameworks that allow system-wide assessment of pandemic plans are therefore needed to understand how core health system functions contribute to resilience in pandemic responses.^12^^,^^13^ Strong health systems and pandemic preparedness therefore reinforce one another and are mutually inclusive.

The objectives of this study were to develop a systematic and comprehensive approach to assessing national pandemic preparedness and response plans from a health system perspective, using existing national influenza pandemic preparedness plans as a basis. We used such plans as they are seen as a blueprint for the pandemic response implementation strategies. Our second objective was to apply our framework to assess national pandemic preparedness and response plans from a health system perspective, identifying included strategies supporting health system performance and potential gaps in health system functions. 

## Methods

We conducted a document analysis of 14 national influenza pandemic preparedness plans submitted to the European Centre for Disease Prevention and Control (ECDC) from countries across Europe (Box 1).^14^ We used the READ (ready materials, extract data, analyse data, distil findings) approach to evaluate the extent to which pandemic preparedness plans include implementation strategies that have been shown to support health system resilience in literature synthesizing lessons from pandemics.^15^ We focused on national influenza pandemic preparedness plans as they are the most common such plans available, and many countries are currently in the process of revising wider pandemic preparedness and response plans.

Box 1 **National influenza pandemic preparedness plans available for analysis and their publication year, March 2024**

Croatia (2005); Finland (2012); France (2011); Germany (2016); Greece (2009); Ireland (2007); Italy (2021); Latvia (2020); Lithuania (2016); Luxembourg (2007); Portugal (2009); Slovakia (2005); Spain (2006); and United Kingdom of Great Britain and Northern Ireland (2011).

Source: European Centre for Disease Prevention and Control, 2024.^14^

We translated 10 of the 14 plans from national languages into English using the DeepL translator, with selected translations validated for accuracy by native speakers.^16^ We excluded Hungary’s national influenza pandemic preparedness plan as a satisfactory translation was not possible.

### Framework for analysis

We adapted the Health System Performance Assessment Framework for Universal Health Coverage (hereafter called the HSPA Framework for UHC), published by the European Observatory on Health Systems and Policies, to guide the assessment of the national plans and to ensure that all relevant aspects of health systems were considered in the study.^6^ The HSPA Framework for UHC links health system performance in core health system functions (governance, financing, resource generation and service delivery) and subfunctions to areas of performance assessment and health system goals.^6^ Our adapted framework links each of the performance assessment areas outlined in the HSPA Framework for UHC to implementation strategies within national pandemic preparedness and response plans that have previously been shown to strengthen health system resilience to pandemics. We identified these implementation strategies by reviewing literature that synthesized lessons from national responses to pandemics, and mapping measures for which evidence exists that the implementation strategies strengthened health system resilience across the core health system functions.^2^^,^^6^ Three assessment areas, namely, stakeholder participation in policy-making, efficient purchasing and availability of health workers, were linked to more than one implementation strategy. For example, availability of health workforce was linked to both ability to scale up existing capacities and recruit additional health workers, and mechanisms to ensure physical, mental and financial support for health workers. We adapted other assessment areas in the HSPA Framework for UHC to allow more appropriate evaluation of the specific implementation strategies included in national influenza pandemic preparedness plans. For example, we split assessment areas jointly covering pharmaceutical and other consumables subfunctions to assess the availability and distribution of pharmaceuticals separately from other consumables, to distinguish between availability of antivirals and availability of personal protective equipment. We also split the effectiveness of the service delivery subfunction into pandemic-specific services and routine services.^8^^,^^17^

### Analysis of preparedness plans

For each of the national influenza pandemic preparedness plans, we used NVivo (Lumivero, Denver, United States of America) to extract information, and systematically linked the implementation strategies identified to assessment areas of the HSPA Framework for UHC to ensure completeness. The degree of inclusion of each implementation strategy was graded using a 3-point scoring system (Box 2), in line with a system used in the Joint External Evaluation. Each implementation strategy scored either 1, 2 or 3 based on the amount of relevant information found in the plan. Where multiple implementation strategies were linked to a single assessment area, we graded each implementation strategy individually and assigned the assessment area an average score across all implementation strategies. Using the example above, if the implementation strategies named ability to scale up existing capacities and recruit additional health workers, and mechanisms to ensure physical, mental and financial support for health workers were scored 2 and 3, respectively, the assessment area called health workforce availability would be scored as 2.5. In the same way, we calculated average scores for each subfunction, function and overall national influenza pandemic preparedness plan. We produced heatmaps using Excel (Microsoft, Redmond, USA) to summarize the results, with traffic-light colouring to indicate average scores.

Box 2 **Description of the scoring system to assess strategies in national influenza pandemic preparedness plans**

One point: No mention of strategy: attributes of a strategy are not in place

Two points: Strategy discussed: attributes of a strategy are mentioned but are in the development stage

Three points: Strategy present: attributes of a strategy are in place

## Results

The framework we developed for assessing national influenza pandemic preparedness plans includes 54 implementation strategies (12 governance, 10 financing, 17 resource generation and 15 service delivery) that improve health system resilience to pandemics linked to the 54 assessment areas outlined in our adapted framework (Table 1).

**Table 1:** Framework of strategies to assess national influenza pandemic preparedness plans from the perspective of health system resilience

Assessment areas, by core health system function and subfunction	Linked strategy
**Governance**	
*Policy and vision*	
Strategic direction in written and traceable form	Develop a clear and timely response strategy and contingency response plans
Quality strategic vision	Develop a comprehensive set of policies, laws and guidelines to indicate how the pandemic response strategy will be monitored and evaluated
Existence of multisectoral collaboration	Establish mechanisms to coordinate within (horizontally) and across (vertically) levels of government, including clear chains of command and responsibility
Quality multisectoral collaboration	Develop plans to support policy implementation, monitoring and evaluation, and training and capacity-building
*Stakeholder voice*	
Political priority for participation	Develop a pandemic cross-party committee or working groups that facilitate political consensus on the response strategy
Stakeholder participation in policy-making	Involve of all relevant stakeholders in policy-making
	Establish clear and transparent communication with stakeholders and relevant populations included in pandemic response plans
	Coordinate pandemic response policies beyond national borders through participation in EU and IHR agreements with relevant actors (e.g. international agencies and other countries’ governments), and regional and global collaborators
*Information and intelligence*	
Collection of relevant information	Establish mechanisms to strengthen monitoring, surveillance and early warning systems, including data collection and data sharing mechanisms between stakeholders, the use of digital tools (e.g. digital dashboards or genomic surveillance), and advanced methods of identifying change in need, access to services and at-risk populations
Evidence-based decisions	Transfer best available evidence from research into policy through mechanisms to generate (or access) and process multidisciplinary scientific information and feed it into decision-making
*Legislation and regulation*	
Documented capacity to legislate	Ensure mechanisms exist for governments to be able to act fast through implementing time bound-emergency legislation (e.g. on lockdowns, purchasing and regulating standards)
Ensured compliance with legislation	Develop mechanisms to establish and maintain public trust in response agencies to support engagement in pandemic response strategies, as well as compliance with emergency legislations
**Financing**	
*Revenue raising*	
Sufficient funds	Develop mechanisms to ensure sufficient funds to meet needs, e.g. having adequate baseline spending on health, earmarking funds for health care, and/or establishing financial reserves for emergency use
Stable funds	Develop mechanisms to ensure stable funds to meet needs, e.g. countercyclical health financing mechanisms, and ability to quickly reallocate general government funds and/or increase levels of public borrowing
Equitable revenue raising	Develop mechanisms to ensure revenue is collected in an equitable way that does not adversely affect poor people
*Pooling*	
Equitable pooling	Establish financial pooling systems that distribute pandemic resources and services equitably across the population (e.g. mechanisms to ensure multiple revenue sources and funding streams organized in a complementary manner, in support of a common set of benefits, and evidence of equitable distribution of financial risk, and/or available policy statements on fragmentation and how to mitigate this fragmentation
Administrative efficiency	Ensure adequate spending on administrative processes to enable harmonized entitlements and service coverage across pools, equalize risk and produce uniform information systems
*Purchasing*	
Efficient purchasing	Adapt purchasing systems to meet changing needs and balance economic incentives
	Develop new and alternative procurement channels to meet changing needs and balance economic incentives
Allocation according to need	Adapt payment systems to reallocate funding to different providers or activities to meet changing needs and balance economic incentives
*Governance of financing*	
Comprehensive coverage	Support universal health coverage and reduce barriers to services by ensuring public knowledge of entitlements and changes to coverage, including health-care services related to coronavirus disease 2019, and/or establishing and broadening of exemptions from user charges
Quality public financial management	Establish transparent and efficient public financial management structures that are responsible for moving resources to the right place at the right time
**Resource generation**	
*Health workforce*	
Availability of health workers	Establish authority to scale up existing workforce capacity by recruiting additional health workers and/or temporarily extending workload of health workers
	Ensure physical safety of and mental and social support for health workers and offer adequate compensation for increased workload and hazardous working conditions
Distribution and mix of the workforce	Implement flexible and effective approaches to using the workforce through subnational mapping of the health workforce, and establishing the administrative authority to reassign health professionals to other areas and providers and/or expanding the responsibilities of health professionals to deliver new types of services
Education of workforce	Provide crisis preparedness and cross-skill training for health workers to support their ability to treat specific or at-risk population groups
*Medical equipment and infrastructure*	
Availability of:	
Medical equipment	Establish pre-pandemic availability of the medical equipment (e.g. ventilators, syringes and primers) required to respond to a pandemic and ensure that there is an agency responsible for organizing emergency supplies and reserves
Medical infrastructure	Establish pre-pandemic availability of the medical infrastructure (e.g. hospitals and outpatient care facilities) required to respond to a pandemic and ensure that there is an agency responsible for organizing emergency supplies and reserves
Distribution of:	
Medical equipment	Establish mechanisms for distributing medical equipment across facilities and households
Medical infrastructure	Establish mechanisms for distributing medical infrastructure across facilities and households
Maintenance of:	
Medical equipment	Ensure routine maintenance for medical equipment is set up, such as scheduled inspections, testing and preventive maintenance for any medical equipment, as guided by the manufacturer’s recommendations and/or the existence of a contracted agency responsible for maintenance and repair of any laboratory machines
Medical infrastructure	Ensure routine maintenance for medical infrastructure is set up, such as preventive and corrective maintenance for electrical, water, sanitation, sewerage or ventilation systems in health facilities
*Pharmaceutical and other consumables*	
Availability of:	
Pharmaceuticals	Establish pre-pandemic availability of the pharmaceuticals (e.g. antivirals and vaccines) required to respond to a pandemic and ensure that there is an agency responsible for organizing emergency supplies and reserves
Other consumables	Establish pre-pandemic availability of the other consumables (e.g. masks and gloves) required to respond to a pandemic and ensure that there is an agency responsible for organizing emergency supplies and reserves
Distribution of:	
Pharmaceuticals	Establish mechanisms for distributing pharmaceuticals across facilities and households
Other consumables	Establish mechanisms for distributing other consumables across facilities and households
*Governance of resource generation*	
Setting quality standards	Establish criteria to regulate standards for the health workforces (education, training, licensing and accreditation systems) and to meet the quality and safety authorization criteria for medical equipment, infrastructure, pharmaceuticals and other consumables, and ensure compliance with standards for manufacturing and procuring pharmaceuticals
Assessment of quality standards	Develop a process for renewing accreditation of educational institutions and health professionals; complete needs assessments and clinical effectiveness measurements for infrastructure and medical equipment; and conduct health technology assessments for quality control inspections, enforcement of marketing regulations, and for supply control mechanisms for pharmaceuticals and other consumables
Planning of resources	Estimate the types and numbers of skills, resources and infrastructure needed to respond to a pandemic and meet health needs
**Service delivery**	
*Public health services*	
Effective delivery of pandemic-related public health services	Implement alternative and flexible patient-care pathways and public health interventions to effectively manage the pandemic disease (e.g. appropriate non-pharmaceutical interventions, find-test-trace-isolate-support services, and vaccination programmes to control and mitigate transmission)
Effective delivery of routine public health services	Implement alternative and flexible patient-care pathways and interventions that effectively maintain access to and performance of essential routine public health services (e.g. early childhood and maternity services and screening programmes); for example, initiating triaging systems and delivering care remotely as needed
Safety of public health services	Ensure safety of patients by introducing additional infection control protocols, including the use of physical barriers to separate confirmed and suspected cases from other patients; the provision of facemasks and other personal protective equipment and sanitation stations for patients and staff; and the increased cleaning of health facilities
User experience	Establish services that meet the needs of the population by introducing mechanisms to collect and value user perspectives on services, and measure user access to services and changes in user utilization
*Primary health-care services*	
Effectiveness	
Effective delivery of pandemic-related primary care services	Implement alternative and flexible patient care pathways and primary care interventions to effectively manage the pandemic disease
Effective delivery of routine primary care services	Implement alternative and flexible patient care pathways and interventions that effectively maintain access to and performance of essential routine primary care services.
Safety of primary health-care services	Ensure safety of patients by introducing additional infection control protocols, including the use of physical barriers to separate confirmed and suspected cases from other patients; the provision of facemasks and other personal protective equipment and sanitation stations for patients and staff; and the increased cleaning of health facilities
User experience	Establish services that meet the needs of the population by introducing mechanisms to collect and value user perspectives on services, and measure user access to services and changes in user utilization
*Specialist health-care services*	
Effective delivery of pandemic-related specialist care services	Implement alternative and flexible patient care pathways and specialist care interventions to effectively manage the pandemic disease
Effective delivery of routine specialist health care services	Implement alternative and flexible patient care pathways and specialist care interventions that effectively maintain access to and performance of essential routine specialist care services
Safety of specialist care services	Ensure safety of patients by introducing additional infection control protocols including the use of physical barriers to separate confirmed and suspected cases from other patients; the provision of facemasks and other personal protective equipment and sanitation stations for patients and staff; and the increased cleaning of health facilities
User experience	Establish services that meet the needs of the population by introducing mechanisms to collect and value user perspectives on services, and measure user access to services and changes in user utilization
*Governance of service delivery*	
Decision-making authority	Establish local coordinators responsible for organizing services to respond to local health-care challenges according to their competencies
Integration of services	Integrate services by introducing and strengthening referral pathways between different providers and levels of care, developing an appropriate regulatory framework and performance and monitoring systems to guarantee the financial, physical and human resources required to create more integrated service delivery systems, and increasing capacity to utilize the private sector to support the provision of services as needed
Quality assurance mechanisms	Maintain quality standards across all services through mandatory professional licences, quality reporting, incident reporting, external audits and inspections

EU: European Union; IHR: International Health Regulations (2005).

Note: assessment areas outlined in the Health System Performance Assessment Framework for Universal Health Coverage were linked to 54 strategies known to support pandemic responses in resilient health systems.

**Fig. 1:**
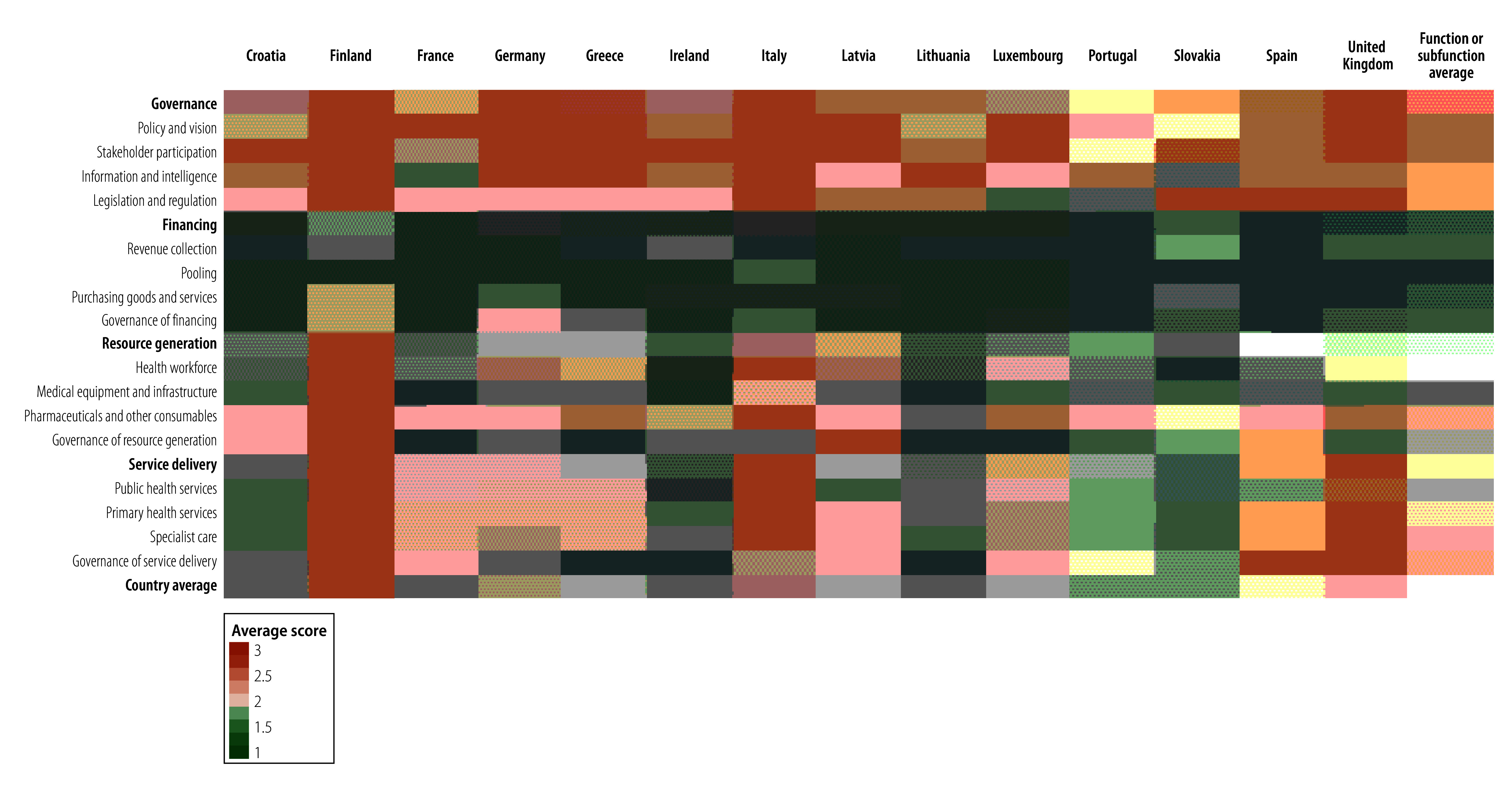
Extent to which strategies to support health system functions and subfunctions are included in national influenza pandemic preparedness plans, by country

Note: Box 2 describes the scoring system.

Fig. 1 shows a heatmap illustrating the extent to which the implementation strategies were found in each national influenza pandemic preparedness plan, covering specific health system subfunctions and functions. For instance, if a subfunction is shown as green, it means that the relevant implementation strategies linked to that subfunction were explicitly present in the national influenza pandemic preparedness plan; on the other hand, if a subfunction is red, it indicates that no mention of the relevant implementation strategy was made for that subfunction.

For example, across national influenza pandemic preparedness plans, the governance function and pharmaceuticals and other consumables subfunction had the highest respective average scores of 2.6 and 2.3, respectively. In contrast, the financing function, and medical equipment and infrastructure, governance of resource generation, and health workforce subfunctions are generally not mentioned in national influenza pandemic preparedness plans (average scores range: 1.2–1.9), and are therefore shown in red shades.

In the following sections we report on the degree to which implementation strategies are included in national influenza pandemic preparedness plans based on the average scores for health system functions and subfunctions using the scoring rubric in Box 2 (i.e. ranging from 1 to 3).

### Governance

Implementation strategies to support assessment areas related to the governance function were commonly found across all national influenza pandemic preparedness plans. The policy and vision and stakeholder voice subfunctions both had a relatively high average score of 2.7 across all countries. Nearly all the national influenza pandemic preparedness plans had clear aims and objectives of a strategic vision, and only two of the plans failed to provide a means to put into action and update implementation strategies according to existing guidance (online repository).^18^ Political participation was also well supported, with most of the plans establishing a national pandemic planning committee with clear chains of command to help facilitate political consensus. Furthermore, most national plans covered involvement of citizens, health workers, civil societies and the private sector in policy decisions and communications. However, for cross-border coordination and international collaboration, only Finland and France include the role of their IHR focal point in coordinating the response.

Implementation strategies to support assessment areas related to the subfunctions information and intelligence, and legislation regulation were less consistently covered, scoring 2.4 on average. While most countries mentioned implementation strategies to share surveillance data with the early warning alert and response system and the Global Influenza Surveillance and Response System, comprehensive implementation strategies to strengthen monitoring systems, such as the use of digital tools and dashboards to capture changes in population health and barriers to access to services, were largely absent. Key governance structures to support the capacity of governments to enforce response measures, such as established special public health legislations, were also missing in five of the national influenza pandemic preparedness plans. Nine plans mentioned mechanisms to monitor and evaluate the response and emergency legislation. However, none of the plans adequately addressed initiatives that enhance public trust and solidarity or support to households affected by emergency legislation.

### Financing

The health system financing function had the lowest average score of 1.2 across countries, with few implementation strategies addressing the performance of its assessment areas. The implementation strategies present covered revenue generation, with Finland and Ireland mentioning the ability to tap into national reserves. Beyond that, however, information related to health system revenues was limited, with only Greece’s national influenza pandemic preparedness plan providing an estimation of funds necessary to manage a pandemic.

For both the purchasing of goods and services and the governance of financing, Finland’s national influenza pandemic preparedness plan scored relatively high (2.3), compared to the averages of 1.2 and 1.3, respectively. This high score is due to the inclusion of provisions on contracting both public and private providers to support efficient procurement of goods and services, as well as the inclusion of a coordinated system to evaluate and adapt purchasing of vaccines. Furthermore, the plans of Finland and Greece clearly state that pandemic-related health services would be provided to patients free of charge. Their plans, as well as the plans of a few other countries, also state the need for governments to introduce price control procedures for vital resources or purchasing authorization mechanisms to reduce barriers to accessing goods and services required during a pandemic. However, explicit reference to public financial management mechanisms that govern the allocation, use and accountability of public funds was largely absent in all national influenza pandemic preparedness plans.

### Resource generation

Few implementation strategies to support the development of the health workforce were found in national influenza pandemic preparedness plans, which resulted in an average subfunction score of 1.9. Implementation strategies to ensure the availability of health workers primarily focused on mechanisms to produce a surge in the workforce through recruitment of medical students and retired workers, as well as short-term crisis training to mobilize, accredit and manage volunteers. Little consideration was given to health workers' well-being: only Finland, Greece and Italy mentioned provision of helplines for psychological support. Furthermore, only Finland’s and Luxembourg’s national plans discussed the ability to reassign health workers to other areas and providers. Implementation strategies to provide staff with crisis and cross-skill training, such as conducting pandemic simulations, was only described in five of the national plans, namely, Finland, Germany, Greece, Italy and Latvia.

Similarly, implementation strategies to support the securing of medical equipment (e.g. ventilators and syringes) and infrastructure (e.g. hospital beds and facilities) only scored 1.6 on average across countries. Instead, all the country plans focused on ensuring overall availability, but nearly all the plans failed to outline the means of distribution and maintenance of resources. Implementation strategies on securing pharmaceuticals (e.g. antivirals and vaccines) and other consumables (e.g. masks and gloves) were covered better (average score 2.3) and detailed the establishment of reserves. Spain’s plan included the distribution of antivirals to each autonomous community based on population size; and Italy identified hospitals and storage facilities from which personal protective equipment could be promptly distributed locally.

Finally, governance of resource generation had a low score of 1.8 on average across the countries. Most countries failed to address planning for the resources required for a pandemic. Only two countries – Finland and Latvia – mentioned mechanisms to safeguard the quality of medical resources and stockpiles. Meanwhile, five countries outlined the need to update resource generation plans as understanding of pandemic protection measures evolved – Croatia, Finland, Latvia, Spain and the United Kingdom of Great Britain and Northern Ireland.

### Service delivery

The average scores for public health, primary care and specialist care service delivery subfunctions were 2.0, 2.1 and 2.2, respectively, mainly because only three countries – Finland, Ireland and the United Kingdom – considered ways to capture user experiences of services during a pandemic. For example, Italy’s plan proposed daily surveys of patients who were under surveillance, or isolating or receiving care at home; as well as knowledge, attitude, practice and belief surveys to identify knowledge gaps, cultural beliefs or behavioural patterns that could facilitate better understanding of community mitigation efforts.

Implementation strategies to support the effectiveness of the public health subfunction were limited: only a few countries identified plans for community use of personal protective equipment, contact tracing, and isolation and support measures. Generally, little consideration was given to maintaining essential public health services, such as screening and immunization programmes. In comparison, triaging systems, remote or online health consultations and facility rearrangement were common implementation strategies for primary and specialist care services in many of the national plans, to ensure ongoing delivery of both pandemic-related and routine services. Additionally, half of the national plans outlined implementation strategies to support the safety of the services being delivered, including strengthening infection prevention and control measures for health workers. These measures included guidance on enforcing the use of personal protective equipment, and increasing cleaning and disinfection requirements, as well as establishing monitoring systems to measure adverse effects of pharmaceuticals.

The governance of service delivery scored 2.0, with just Finland, Spain and the United Kingdom providing clear implementation strategies across all performance assessment areas. These were also the only countries, alongside Italy, that included pre-established integration between primary and specialist care services in their plans to help tackle a surge of patients. Capacity to monitor the quality of services was also only covered in five national plans – Croatia, Ireland, Latvia, Lithuania and the United Kingdom.

## Discussion

In this study we evaluated the extent to which pandemic preparedness plans in Europe consider the entire health system. We found that these plans often missed opportunities to address major health system areas, especially financing, distribution of physical resources and planning of resources. We believe that the framework we used can be adapted systematically to evaluate the coverage of all aspects of a health system in pandemic preparedness plans.^6^

Pandemic risk management and preparedness, traditionally guided by national influenza pandemic preparedness plans, can and should help to make health systems more resilient to shocks.^3^ While many health systems in Europe eventually found ways to respond to COVID-19, those countries with more robust initial capacities found it easier to initiate and resiliently manage the pandemic response while maintaining health system performance and continuity of care.^1^^,^^2^ Finland’s national plan broadly includes implementation strategies across the health system (with the exception of financing) to strengthen health system resilience. While we cannot attribute Finland’s successes directly to its national influenza pandemic preparedness plan, we note that the COVID-19 pandemic in Finland was not as extensive as in other countries, with infection levels five times lower than the EU average, limited disruption to health-care services and relatively smaller adverse effects on the economy.^19^^,^^20^ This success may be in part due to the comprehensiveness of implementation strategies, such as the Finnish national influenza pandemic preparedness plan.

Therefore, countries should recognize the importance of investing in health system capacity to strengthen pandemic preparedness. Our study of national influenza pandemic preparedness plans suggests some areas are neglected. Therefore, implementation strategies to support health system performance in these area should be considered as countries revise their influenza-specific and wider national pandemic preparedness and response plans to help strengthen the overall resilience of the health system to future pandemics.

In particular, the few implementation strategies to support health system financing in the national plans (even if financing plans exist elsewhere) suggests poor financial planning and a lack of mechanisms that can be used to respond to sudden needs; for example, to raise, reallocate, and spend emergency funds, or alternatively to dedicate funds before an event that can be used to build capacities in advance and strengthen preparedness more widely. While financial aspects may be addressed elsewhere in other government plans or policies in some countries, a clear reference to these documents in the national influenza pandemic preparedness plan would enable better coordination and more appropriate designation of financial movements. However, none of the national influenza pandemic preparedness plans we reviewed had such a reference. Our findings point to the need for countries to re-evaluate their existing financing arrangements considering their current experiences. These experiences can be included in the national influenza pandemic preparedness plans to prepare for future pandemics, as well to improve health system efficiency and equitable access to health care.^21^

Our study has several limitations. First, the scope of this study was limited to publicly available national influenza pandemic preparedness plans. Obtaining access to all national influenza pandemic preparedness plans across Europe would provide a better understanding of the current gaps in implementation strategies to strengthen health system resilience. Furthermore, we assumed that national influenza pandemic preparedness plans guided the COVID-19 pandemic response, but we do not know to what extent they were used in practice. For example, our results indicate that implementation strategies to support health system governance were well documented across the plans, but this aspect was not very evident in countries’ responses to the COVID-19 pandemic. While the lack of this information could be perceived as a limitation, planning documents are meant to guide action, which depends on other governance-related capabilities that were not within the scope of this study.

Second, the implementation strategies we linked to assessment areas are not exhaustive or context-specific and were informed primarily by literature specific to the COVID-19 pandemic response. Implementation strategies less evident in national influenza pandemic preparedness plans, such as how to distribute and set quality standards for resources, may be business-as-usual processes that national planners outline in other operational documents.

Third, the 3-point scale used to score the inclusion of implementation strategies only provides a basic level of assessment. However, the system is similar to other scoring systems used for comparable purposes and is intended only to provide an overview. In addition, some subjectivity in scoring may be present; we addressed this issue by using multiple scorers for each national influenza pandemic preparedness plan.

In conclusion, our findings provide important insights into the blind spots of pandemic preparedness planning documents. Many countries are now in the process of reviewing their national influenza pandemic preparedness plans, considering WHO’s initiative to focus pandemic preparedness plans on the establishment of resilient responses rather than on tackling specific pathogens. Going forward, it is important to ensure that these plans adequately account for health system functions and support resilience in the face of future pandemics.
